# Making Conscientious Decisions: Engaging in Venous Leg Ulcer Self-Management Following Nurse-Led Patient Education

**DOI:** 10.1177/10497323241285692

**Published:** 2024-10-28

**Authors:** Paul Bobbink, Géraldine Gschwind, Philip Larkin, Sebastian Probst

**Affiliations:** 1 Geneva School of Health Sciences, 128870 HES-SO, University of Applied Sciences and Arts, Western Switzerland, Geneva, Switzerland; 2 Faculty of Biology and Medicine, University Institute of Higher Education and Research in Healthcare, University of Lausanne, Lausanne, Switzerland; 3 Wound Care, Outpatient Surgery Unit, 60534 Hôpital du Jura, Delémont, Switzerland; 4 Palliative and Supportive Care Service and Institute of Higher Education and Research in Healthcare, Department of Palliative and Supportive Care, Hôpital Nestlé, Lausanne University Hospital and University of Lausanne, Lausanne, Switzerland; 5 Care Directorate, 27230 University Hospital Geneva, Geneva, Switzerland; 6 College of Medicine Nursing and Health Sciences, 37436 University of Galway, Galway, Ireland; 7 Faculty of Medicine Nursing and Health Sciences, 22457 Monash University, Clayton, VIC, Australia; 8 Faculty of Medicine, University of Geneva, Geneva, Switzerland

**Keywords:** therapeutic patient education, venous leg ulcer, grounded theory, self-management

## Abstract

Venous leg ulcers (VLUs) provoke multiple symptoms and impact individuals and society as a whole. Their treatment and prevention strategies require individual’s involvement in self-management strategies. Insufficient knowledge with regard to prevention, management, and treatment has been identified as a critical factor related to VLUs and their recurrence. Therapeutic patient education (TPE) proposed as part of a management strategy for this population provides unclear benefits regarding wounds healing or prevention of recurrence. The aim of the study was to develop a theory explaining how individuals with a VLU experience an individualized nurse-led TPE program regarding self-management strategies. The constructivist approach of Charmaz to the grounded theory method was used to develop the theory. A total of 26 individuals contributed to the co-construction of the theory through face-to-face or telephone semi-structured interviews. Data analysis and data collection occurs simultaneously with a comparative process to reveal the conceptual categories, apply theoretical sampling, and define theoretical saturation. The theory of “Conscientiously Engaging in Self-Management” was co-constructed with the participants encapsulating four categories: “Being influenced by my own story,” “Being personally informed,” “Making conscientious decisions to engage in self-adapted management strategies,” and “Integrating a conscientious way of living.” This theory highlights individuals’ voices and stories toward their journey of VLU self-management taking contextual factors into consideration. This new theory offers new knowledge about implementation of self-management strategies for individuals living with a VLU and will inform clinical practice and contribute to the development of targeted interventions.

## Introduction

Venous leg ulcers (VLUs) reflect the highest grade of chronic venous insufficiency (Lurie et al., 2020) and are defined as an open skin lesion of the lower leg affected by venous hypertension (O’Donnell et al., 2014). VLUs represent the most prevalent chronic lower leg wound (Guest et al., 2020) with an estimated prevalence around 0.32% (Probst, Saini, et al., 2023). VLUs estimated healing time in European countries is 13 months (Gethin et al., 2021) and their recurrence rate rises to 60 % at 1 year (Finlayson et al., 2015). Therefore, VLUs represent an important burden not only for persons concerned but also for healthcare systems (Phillips et al., 2020).

VLUs have a broad impact on health-related quality of life (HRQoL) (Smith et al., 2023) and can result in physical impairment such as fatigue, wound-related pain (Leren et al., 2020; Probst, Gschwind, et al., 2023), or exudate, all of which contribute to a decrease in perceived HRQoL (Folguera-Alvarez et al., 2022; Lin et al., 2022). Having a VLU results in social restrictions (Phillips et al., 2018) and increases guilt for the person concerned when imposing the burden of their condition on family or friends (Klein et al., 2021). Additionally, individuals living with a VLU are at risk for other chronic conditions (Kelly & Gethin, 2019), frequently multiple in nature (Gethin et al., 2021) and with an increased risk of death (Prochaska et al., 2021). Specific contributory factors leading to VLUs remain unclear. However, age, a history of deep venous thrombosis, family history, reduced mobility, or obesity could increase the risk of developing a VLU (Meulendijks et al., 2019, 2020).

To prevent, manage, and treat recurrences of VLUs, various guidelines recommend treatment requiring self-management skills (Tan et al., 2019). These include, among others, wearing compression therapy, practicing specific exercises, and following a specific diet (Franks et al., 2016). While compression therapy is supported by a high level of evidence for initial treatment or prevention of recurrence (Dahm et al., 2019; Nelson & Bell-Syer, 2014; O’Meara et al., 2012; Patton et al., 2023), other self-management recommendations, such as exercise for lower leg, including ankle resistance (Jull et al., 2018; Qiu, Osadnik, et al., 2022; Turner et al., 2023), or adapted nutrition (Barber et al., 2018), have lower levels of evidence.

Evidence suggests that the etiology of VLUs seems to be misunderstood by those affected (Boxall et al., 2019; Chase et al., 2000; Phillips et al., 2018; Qiu, Team, et al., 2022) as close to 40% of individuals did not understand the “venous” aspect of their wound (Edwards et al., 2002). As a consequence, engaging in adapted self-management strategies seems questionable.

A close relationship between nurses and individuals living with a VLU has been described, highlighting the importance of their regular contact, offering the opportunity for a unique relationship (Klein et al., 2021). To address these challenges, multiple therapeutic patient education (TPE) programs have been developed by nurses using various modalities and content (Bobbink, Pugliese, et al., 2020). Nevertheless, the effectiveness of TPE or how it works remains unclear when applied to individuals living with a VLU (Gomes et al., 2024; Shanley et al., 2020; Weller et al., 2016) and warrants further investigation. Therefore, this study sought to understand how individuals with VLUs experience an individualized nurse-led patient education program regarding self-management of VLUs.

## Methods

This study is a sub-study of a larger randomized controlled trial (RCT) (NCT04019340) to evaluate the effectiveness of nurse-led intervention for patients with VLU in terms of patient knowledge/therapy adherence and to measure the impact of this intervention on wound size reduction and its evolution over time. The protocol for this study, a constructivist grounded theory methodology (CGTM), is reported elsewhere (Bobbink, Larkin, et al., 2020). The quality criteria of the proposed constructivist grounded theory (CGT), including credibility, resonance, originality, and usefulness, are discussed in the strengths and limitations section.

### Design and Philosophical Framework

To generate the theory, a constructivist approach to the grounded theory method (GTM) was employed (Charmaz, 2014). The CGTM arises from a philosophical perspective that assumes people are involved in the construction and interpretation of realities through their own perspectives (Bryant & Charmaz, 2019). This approach allows an active engagement by the researcher, or research team, throughout the entire process of investigation and theory generation (Singh & Estefan, 2018), resulting in a contextualized co-constructed theory (Bryant, 2017).

### Study Team

The study team comprised PB, a PhD student in nursing specializing in wound care and novice qualitative researcher, supported by two senior researchers PL, a professor of nursing with expertise in qualitative methodology, and SP, a professor of nursing with expertise in wound care, qualitative methodology, and CGTM. During the coding phases, GG, an MSc degree nurse specialized in wound care, joined the team to enhance abstraction during coding, category generation, and theory development.

### Sample, Intervention, and Study Site

A convenience sample of individuals who engaged in an individualized education program, were diagnosed with an open VLU, were aged over 18 years, and provided written consent were recruited for this study. The intervention was constructed using an interprofessional approach, including a nutritionist, physiotherapist, and nurses specialized in wound care, and was carried out individually during five meetings by a clinical nurse specialist in wound care including the themes of compression therapy, nutritional support, and physical activities such as stretching and ankle exercises. This tailored intervention was supported by a specific brochure, built across interprofessional expertise and containing specific explanations about VLUs etiology and self-management strategies (Probst et al., 2020). The study was conducted in two outpatient clinics at university hospitals and one outpatient clinic specialized in wound care in French-speaking Switzerland.

### Data Generation

To obtain access to participants’ context and meanings they attribute to their experiences, data were generated through 26 semi-structured audiotaped interviews conducted by PB from April 2020 to July 2023. Due to the COVID-19 pandemic and the need to prevent risk for participants, data generation began with telephone interviews (*n* = 17). Based on the changing pandemic situation and participant preference, subsequent interviews were also held face to face (*n* = 9) in a private room at the university or at home. The mean interview time was 48 min (min: 32 minutes, max: 75 minutes) resulting in close to 20 hours of recorded interviews. Due to a technical issue, one interview was interrupted at the early stage (<2 minutes) of recording and only field notes could be used for data generation. Interviews were conducted in French, expect for one carried out in English according to participant preferences.

The initial interview guide included questions such as “You followed five consultations with a tissue viability nurse. What was your experience of these consultations?”, “Could you describe what it changed in your day?”, and “Tell me, how do these sessions influence your journey living with venous leg ulcer?”. This guide was designed to maintain sufficient openness to achieve a comprehensive in-depth understanding of the research field (Charmaz, 2014) and has been previously published (Bobbink, Larkin, et al., 2020).

As a symbolic interaction, interviewing places both the researcher and the participant in the field of inquiry (Miller & Glassner, 2021) allowing for an active co-construction of the theory through a conversational approach (Charmaz, 2014). As an important point in GTM (Tarozzi, 2020), data generation and analysis occurred simultaneously. To promote theoretical sampling and using the flexibility of semi-structured interviews, the interview guide evolved during interviews to better understand participants’ concerns to inform aspects of emerging categories and improve confrontation of data between participants to advance the construction of the theory. Therefore, during the last three interviews, the preliminary model of the theory was presented at the end of the interviews and discussed with the participants. Both males (12) and females (14) with a wide range age, a variety of sociodemographic variables (Table 1), multiple life experiences, and points of view contributed to the development of the theory including both individual and shared experiences. Twenty-six interviews were considered sufficient to obtain theoretical saturation, defined as finding no new data supporting theoretical insights or new properties of the categories (Charmaz, 2014).

**Table 1.** Participants’ Main Characteristics.

Sociodemographic (*n* = 26)	
Age, mean (SD) (min–max)	62.3 (13.6) (35–83)
Female	63.1 (15.7) (35–80)
Male	61.3 (11.4) (46–83)
Sex, *n* (%)	
Female	14 (53.8)
Male	12 (46.2)
Civility, *n* (%)	
Single	5 (19.2)
Married	7 (26.9)
Divorced	9 (34.6)
Widowed	5 (19.2)
Education, *n* (%)	
Mandatory school	5 (19.2)
Maturity or equivalent	4 (15.4)
Professional diploma or equivalent	10 (38.5)
University degree or equivalent	7 (26.9)
Occupation, *n* (%)	
Professionally active	11 (42.3)
Retired	12 (46.2)
Disability insurance	1 (3.8)
Other	2 (7.7)
Annual salary, *n* (%)	
<25,000 CHF	2 (7.7)
25,001–50,000 CHF	11 (42.3)
50,001–100,000 CHF	8 (30.8)
>100,000 CHF	3 (11.5)
Doesn’t want to answer	2 (7.7)
Health information (*n* = 26)	
Participant with a first VLU, *n* (%)	9 (34.6)
Participant with a recurrent VLU, *n* (%)	17 (65.4)
Number of recurrences, mean (SD) (min–max)	2.6 (2.7) (1–12)
Diabetes, *n* (%)	6 (24.0)
BMI, mean (SD) (min–max)	32.6 (8.5) (17.4–51.8)

### Data Analysis and Interpretative Stance

First, interviews were transcribed verbatim by PB or a professional transcriber. During initial coding, data were fractured into the smallest section of text referring to its meaning (Tarozzi, 2020) using MAXQDA. During this phase, a significant number of codes using gerunds were generated. At this phase, the interviews and coding process were regularly discussed and reviewed between PB and SP. PL provided methodological support during initial coding to focus on meanings. During focused coding, the use of diagrams with pen and paper, and later on VISIO become useful tools to better understanding the data, ensuring constant comparison among data, codes and the development of categories and their connections. During the process, all the codes were transferred to an Excel sheet and printed out and categories were checked against codes and related verbatims. Bringing back to paper and pen allows to prevent the mechanical activity in coding related to computer-aided qualitative data analysis and support the reflexive process. During this step, discussion between PB and GG facilitated intersubjectivity, allowing for the emergence of similarities and discrepancies between data and codes to promote reflexivity and abstraction.

Finally, to promote constant comparison, the categories were revised listening again to the audiotaped interviews, and some random interviews were critically discussed with PL regarding the evolving theory. During this phase, participants guidance, as well as French- and English-speaking nurses with various academic degrees recruited mainly through conferences and practicing in the field of patient education or wound care provided feedback on the emerging models to strengthen data analysis and its usefulness for application to clinical practice. PB participated in grounded theory workshops to improve methodological understanding and pursue data analysis. Awareness of existing and new published evidence in the field supported both abstraction and guiding interview questions. However, the resultant CGT is presented independently. Memos documented the process regarding the interviews or thoughts about the coding process, reflecting the need of future adaptation of the interview guide and including initial naming of the categories or why codes were merged together. Finally, a second research assistant and PhD student with experience in qualitative research read through all the transcripts and presented results and supported the representativity between data and categories.

### Coding and Translation Process

Coding and translation present challenges in CGTM, as they involve more than just linguistic tasks and can offer analytical advantages for understanding textual data (Tarozzi, 2019). Therefore, initial coding was conducted in the language of the interviews, and the progression to refined categories occurred gradually, using translation as an analytical tool to achieve abstraction. The verbatims presented in the manuscript were translated from French to English by PB and reviewed by GG and a research assistant to ensure fidelity to their original meanings. Supplemental File 1 provides the interview numbers, original quotes, and their translation.

### Ethical Considerations

This research project was approved by the Ethics Committee of the Canton of Geneva (CCER: 2019-01964). Prior to the study, participants received oral and written information detailing the objectives of the study, the procedures involved, liability insurance, and data confidentiality. Written consent was mandatory prior to data collection.

## Toward a Contextualized Grounded Theory of Engagement in VLU Self-Management

Drawing from the rich in-depth life experiences of 26 individuals, multiple perspectives supported the construction of the theory entitled “Conscientiously Engaging in Self-Management.” This current contextualized theory explains how individuals living with a VLU conscientiously engage in self-management strategies following an interprofessional nurse-led educational session. It illustrates the experiences within these educational sessions and shows how individuals conscientiously decide to implement adapted self-management strategies into their daily lives, under influence of contextual mediators and aiming for a better future.

**Figure 1.**
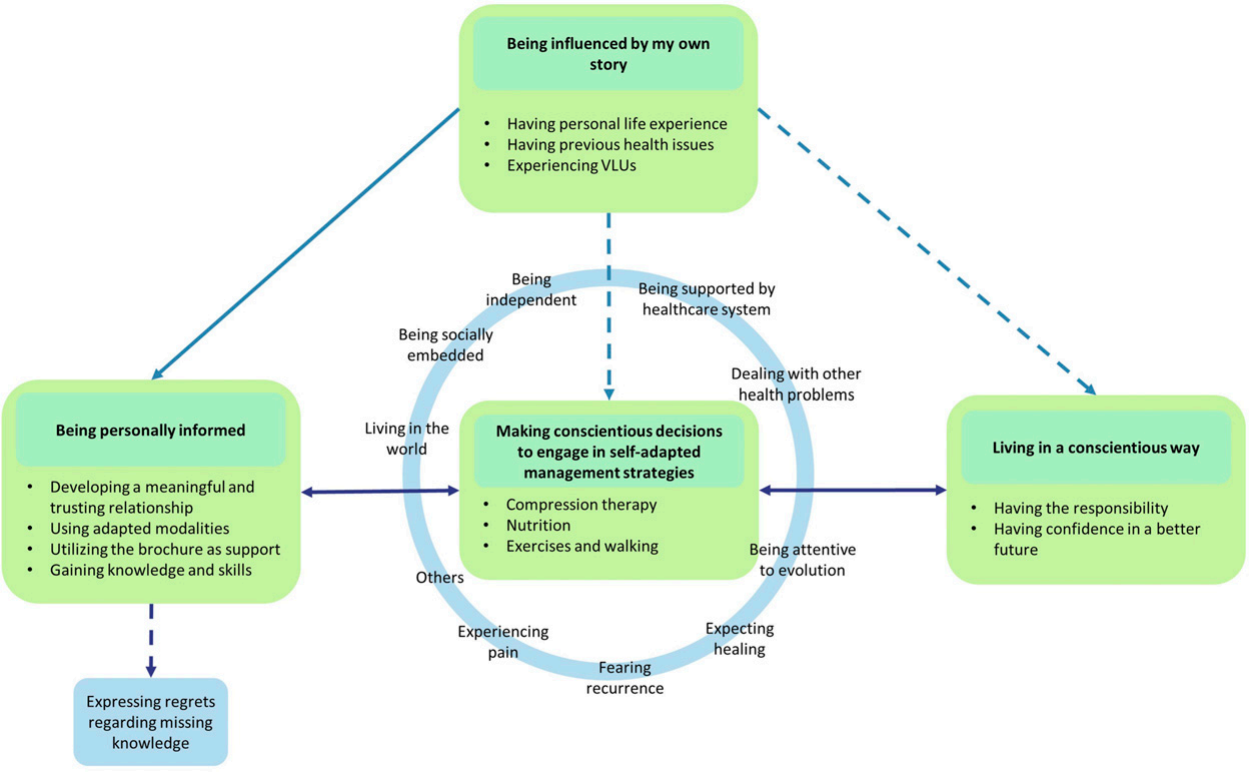
Conscientiously engaging in self-management.

The theory contains four main categories: “Being influenced by my own story,” “Being personally informed,” “Making conscientious decisions to engage in self-adapted management strategies,” and “Integrating a conscientious way of living.” These categories will be outlined below. However, it is important to note that the category “Being influenced by my own story” influences the entire process (Figure 1).

### Being Influenced by My Own Story

The category “Being influenced by my own story” is pivotal throughout the entire journey (i.e., process of decision making), as it highlights values and beliefs influenced from the individuals’ past. It encompasses sub-categories of having personal life experiences, having previous health issues, and experiencing VLUs. Having personal life experiences is an inclusive sub-category that illustrates how individuals with a VLU encountered or coped with various life events shaping their approach to living with and experiencing a disease. For example, one participant explained, “As I always say, we all have to die one day!” (1710), whereas others believed that since “I reached a certain age, things happen and you just have to accept it, there is nothing else you can do” (2030). Beliefs varied significantly among individuals. Some wanted to see the benefits to believe in them, while others relied on prayer to navigate through a challenging day.

Having previous health issues is an integral part of individuals’ stories, shaping their relationships with healthcare providers and presenting challenges in self-management, especially concerning chronic conditions. For instance, individuals living with Factor V Leiden disease, who have had to wear compression therapy for decades to prevent thrombosis, face a high risk of death when deciding not to engage in self-management strategies. Another example involves strategies developed to overcome depression related to children loss during pregnancy or explaining the challenges faced in overcoming drug addiction.

I used to be a drug addict and it took me years … to stop using heroin, so … hum …. I managed to stop on the day … when … I said to myself, well now it’s either in two months I’m “cold” or I’m doing something. Before I didn’t see the point either, and then no one could made me stop …. Before people could have told me anything I wouldn’t have stopped anything. (1807)

These experiences shaped individuals’ health experiences, highlighting abilities of individuals to making decision to engage in lifestyle changes.

Another component encountered was the participants’ experience with previous wounds/VLUs. Table 1 highlights its heterogeneity. Some individuals had no prior experience with a VLU resulting in a lack of knowledge about VLU and unawareness of their existence. They expressed sentiments such as: “For me an ulcer is internal, it’s in the stomach” (3012) or “The thing is, first of all, what I have, and what I am going through, I didn’t know that this exists … So I had a shock to know I found myself in such a situation where I know nothing about” (2030).

Based on their life experiences, they believed in a spontaneous healing without complications.

Other participants were facing up to 12 recurrences and had been living with VLUs for the past 15 years, closely comparing their experience to a handicap. Consequently, they acquired skills and knowledge over time. One participant clarified, “Because with time, I don’t want to say that I’m an expert, but I realise two or three things” (3017). They illustrated this ability by explaining the steps of wound healing, successfully closing a VLU, or being able to seek information independently. This previous knowledge about VLUs and acquired skills enhanced their ability to reflect on why recurrences occurred and raised new questions.

### Being Personally Informed

Being personally informed refers to nurse–patient relational experience and the personalized approach of implementing the educational session. This category comprises sub-categories of developing a meaningful and trusting relationship based on nurse and participants characteristics, using adapted modalities, utilizing the brochure as support, and gaining knowledge and skills. The tailored interprofessional educational program consists of five nurse-led meetings and a brochure. According to participants’ experiences, the combination of both was adapted in an individualized manner depending on participants, nurses, and context allowing for a “sympathic time” and finding answers to their questions. One individual explains, “I had to have this kind of … of … of meeting to get answers to some really simple questions, like: is it useful to walk? Or is it bad for the wound?” (1070).

Developing a meaningful and trusting relationship was an important part of the educational process, as participants received a lot of information from the nurse. Participants emphasized the importance of time to get to know each other and partnering together through their disease. Finding individualized solutions based on participants’ experiences and needs was key to making further decisions to engage in self-management. For example, being listened to by the nurse and discussions with someone knowledgeable were helpful starting points for building a relationship. One individual stated, “but what’s more, when someone knows about [ulcers] and you can talk with, well that’s a relief” (1809). Another participant illustrated the need to construct the relationship: “Well, now we have managed to tame each other and now we adore each other” (3017). The nurse’s characteristics of clinical expertise, humanism, sympathy, reassurance, approachability, and genuine interest in individuals’ stories helped them acknowledge the nurse as a pillar and conduit for mutual strength in a positive atmosphere. This meaningful relationship facilitated effective communication:

I think that things are going well with her because it’s a two-way process, so she suggests things to me and I, I, well, communication is going in a two-way direction, so she tells me things, I take them on board and I tell her hum, hum, well, I don’t refuse to do things, but let’s say I give her feedback on what I see. (3017)

Nurses were able to adjust and adapt the session’s modality to address an individual’s needs, for example, employing specific and detailed information, covering the content of the brochures in order, searching for specific videos on the internet, or using themselves as examples for compression stockings or exercises. One individual stated:

She explained to me the problem I was having, in little words I could understand. Because for me, all these terms were, at first, difficult, I didn’t understand … the message they wanted to convey. But then I understood, and she explained it to me with … but really with drawings. (3028)

However, adapting to each individual remained a challenge, and some individuals undertook the “expected” patient’s role during the educational session.

The utilization of the brochure as a support tool varied from session to session, depending on both the individuals and the nurse. Individuals with lower levels of knowledge about their VLU considered the brochure “a bible in … in its context” (3012), deriving significant understanding due to pictures and diagrams. However, younger participants may have perceived the brochure as possibly designed for an elderly population and so unsuitable: “Without wishing to dissociate myself from the relevance of this brochure, in fact it is mainly aimed at people much older than me … and then … and then that’s it, I just had a look” (1070).

Nonetheless, everyone read through it to acquire the knowledge or to be able to communicate effectively with the nurse. The subsequent reuse of the brochure was dependent on individuals, with some taking note inside, sharing it with their partner, extracting regular ideas for a specific dietary or exercise routines, and using it as an aide-memoire when implementing self-management strategies. One participant summarized the use of the brochure during the educational process:

These are the three elements that really impressed me in this brochure, this complete and extremely practical systemic approach, which can be implemented after a few explanations … which were given to me by [study nurse name] and the team. (3031)

Following these sessions, most individuals reported increased knowledge and skills regarding etiology of VLUs and various management strategies. They learned about the reasons behind and the proper methods for wearing compression therapy, as well as how to prepare an adapted diet and use exercises in their daily life. Understanding the relationship between their wound and venous insufficiency enabled them to grasp the importance of compression therapy and provided them with new motivation to participate in activities such as specific ankle exercises. Understanding the risk of recurrence facilitated long-term changes. However, the extent to which this sub-category influenced individuals varied. Some individuals, in connection with their own story and experience of VLUs, did not acquire new knowledge:

Well, for me, I’ve already learnt a few things, um … for me personally, what’s important now is to … hum … apply what I already knew and what I learnt during this study um … in terms of prevention so that there isn’t another relapse, well that I don’t have another wound. (3042)

### Making Conscientious Decisions to Engage in Self-Adapted Management Strategies

Making conscientious decisions to engage in self-adapted management strategies constitutes the essential components of this constructivist grounded theory. These categories are interconnected by a reciprocal relationship, each influencing the other.

Making conscientious decisions involves specifying how, when, and why individuals make decisions in a continuous process, influenced by their personal experiences, the education sessions, the knowledge, reassurance and skills gained from education sessions, as well as contextual factors, to find an equilibrium. The decision-making process was variable and sometimes relatively fast and easy: “If you tell me you have to wear them every day, well I will wear them every day. If you tell me you have to wear them every two days, we’ll I will wear them every two days. I am stupid and disciplined” (3012), or take time “Well, with me you have to plant the seed and after a while it grows” (3017).

Understanding the etiology of the VLU enabled individuals to act and make choices about the components of the intervention that best fit their everyday lives. While some participants acknowledged following all nurses’ advice and guidance, others only partially followed the rules. Nevertheless, decision making was a conscientious process, readjusted and contextualized based on the expected benefits of risks perceived by each individual considering their overall context. One participant explains, “I don’t have a choice, the best thing is to do what you have to do when you have to do it, but from time to time allow yourself a few libertie*s*” (1809), thus highlighting the importance of making individual decisions in their self-management journey.

Engaging in self-adapted management strategies reflects how and why individuals implement and adapt their actions/strategies of self-management. This category was closely interrelated with the decision-making process, present during and after the implementation of self-management strategies. Finding a comfortable way to engage is crucial to support changes over time. Individuals emphasized the significance of making step by step changes, gradually doing more each day to achieve long-term behavior change which appeared essential for many of them. This engagement was continuously adjusted, challenged, and influenced, positively or negatively by the contextualized factors. Making conscientious decisions to engage in self-adapted management strategies involves considering the main components of the intervention, which included compression therapy, walking, exercises and nutritional support, and progressive implementation. One individual highlighted, “I’ve already made efforts in other areas, but I’m finding it hard to make all these efforts at the same time, and that’s part of the problem” (1070).

Most participants understood the expected benefits of applying compression therapy for healing and preventing recurrence. However, this understanding did not always result in consistent and rigorous application of compression devices. Due to various contextualized mediating factors, such as being supported by healthcare system, individuals often found strategies to support and adapt compression therapy to their needs. These strategies included not wearing compression therapy on some evenings when going out, using specific devices to aid in putting on stockings, wearing adjustable “wrap” devices, and wearing an old pair of stockings during water sports. Some individuals experienced a rapid reduction in symptoms, such as no longer needing cold showers at night, which enhanced their adherence to compression therapy. On the other hand, after a conscientious decision-making process, some individuals decided not to wear compression therapy, even though it increased the risk of non-healing or preventing recurrences:

Well there are also the compression stockings, after all I know that logically I should be wearing them for life, which I also found very hard to accept …. And then … normally I’d have to wear—I’ve got a wound on my right leg, and then several years ago I also had a wound on my left leg, which closed up by itself, and then I’d have to wear a stocking on my left leg as well, but that’s something I find very hard to accept too …. (1807)

Individuals perceived exercise or walking as interrelated, enabling choice between them based on the activities of their daily lives. For instance, some individuals walked a lot for work or for other personal reasons, such as visiting the cemetery. Additionally, they adjusted their exercise routine based on factors like the weather. They might lower or avoid exercises and increase walking in good weather and increase exercise and avoid walking on days when the roads were slippery. Engaging in specific exercise designed for promoting blood flow return was a novel concept for some participants, who previously believed that immobilization was preferable. However, upon experiencing the direct benefits of exercises on symptoms such as heavy legs, they became more disposed to continue exercising. Due to the simplicity of exercises, like leg movements, individuals found it easy to incorporate them into their daily routine, such as while watching TV. Moreover, they found it feasible to adjust the difficulty level of exercises based on their own experience, comfort, or other health conditions:

Let’s say …. I … the problem was that I had a …. I had a splint that I’d had for almost a month, you see!? So I couldn’t move as much as I used to and then um … so the exercises, so I did them let’s say with the splint, on the left side …. I did right, left, um … forwards and backwards, and then … well I couldn’t force it like … like I would have liked …. (1710)

Adapting diet was a complex and delicate aspect of implementation. Implementing actions to improve nutrition faces multiple mediators requiring engagement in changes: “And eating very healthy is an activity in its own right … if you don’t mind me saying so” (1070) or:

If I had twice as much time, we'll I would chop the vegetables, cook them, and so on and so forth. But honestly, hum … no, we don’t have much time …. (3012)

Others improved their consumption of fruits and vegetables, changed their behaviors, and stopped drinking alcohol. One individual elaborated, “But in any case, it’s given me a wake-up call in that respect, that it’s really worth paying attention and … and that’s important even if it’s not immediately apparent … but it has its importance in the … for the skin” (3042).

### Being Influenced by Mediators

Making conscientious decisions to engage in self-adapted management strategies always takes place under the influence of mediators. The mediators of the process comprise several influential contextual factors, either through sustaining or impeding the self-adapted implementation of self-management strategies and health behavior changes (Figure 1). These factors include, among others, “living in the world,” “being independent,” “dealing with other health problems,” “being attentive to evolution,” “fearing recurrence,” “expecting healing,” and “experiencing pain.” These are illustrated below.

Living in the world illustrates how participants interact with their environment as a whole, highlighting, for example, the impact of weather, or work on the decision-making process. One participant noted, “Well at work, for example, in the drink machines, you only get lemonade … you only get lemonade with 10 milligrams of sugar per deciliter … so there is nothing else” (1807). Experiencing COVID-19 lockdown was also a mediator, reducing, on one side, the possibility to attend social meetings or restaurants but conversely improving the ability to find solutions to gain self-management skills. This mediator living in the world also included the possibility to adapt their environment to implement strategies to take care of their leg. For example, one individual raised the legs of the bed to improve blood flow return.

Being socially embedded refers to the importance of living in interaction with the family or society at large. It underscores the duality of receiving care and providing care for others. For instance, experiences like having a baby, sharing a meal with a friend, being supported by a loved one, or taking care of the neighbor’s dog served as motivators for individuals to engage in activities such as going for a walk, acting as external motivators beyond coping with the disease. However, some individuals faced challenges such as negative perceptions of their legs with compression therapy or difficulties in managing self-care strategies when going to work.

Being independent reflects the importance individuals place on managing their situation autonomously, without depending on others to manage their disease. As an illustration, applying compression therapy on oneself could be challenging and might lead to its rejection if no solution was found. However, this factor also had positive aspects, as participants learned to change their dressings to suit their lifestyle, thus becoming more independent. One individual explains, “You have to keep your independence, and you must … you mustn’t let yourself slump in your armchair all day either” (1109).

Being supported by healthcare systems acts as a mediator that either supports or impedes the adoption of best care practices, depending on individuals’ experiences with healthcare systems. For instance, having frequent changes in healthcare providers in homecare or experiencing challenges when going to the hospital sometimes promotes individuals to take care of themselves independently. However, being encouraged by the healthcare team, receiving from an interdisciplinary team, or knowing the possibility of getting support for compression therapy within their healthcare structure proved helpful in implementing strategies.

Dealing with other health problems was the foremost mediator in the process as some components of the intervention could not be carried out by individuals due to their current or previous health experiences. Therefore, individuals adapt their self-management strategies:

Unfortunately, I can’t do some exercises like everyone else because I’ve had two accidents … so on top of that I no longer have any tendon in my arms, so that means I’m still handicapped for quite a few things in my arms and that doesn’t allow me to pull a rubber band or do that, but on the other hand everything I can do with my ankles, knees, feet and all that, I showed her [the nurse], she told me that I proceed correctly. (1809)

Conversely, another individual living with a hereditary hypercoagulability noted, “It’s not as if I could live without wearing compression stockings, for the simple reason that it’s not bearable. Because I think she [the study nurse] told you I have Leiden syndrome that’s why no” (1109).

Being attentive to evolution is situated between an expectation of healing and fearing recurrences with a direct impact on perceived benefits. This mediator encompasses signs and symptoms individuals can assess in themselves, allowing them to act accordingly. For example, perceiving positive evolution in wound healing and associated symptoms positively influenced their confidence in self-management. One individual explains, “I can already see that if you can eat a bit of everything, the healing process improve, going much more smoothly” (2050). However, experiencing a lack of progress, VLU recurrences, or negative symptoms related to self-management raised questions about how to implement them in their own context.

Expecting healing, defined as hoping to close of the wound, was a strong motivator for engaging in self-management. However, for some individuals living with VLUs over years, this outcome was less motivating. One individual argues:

But I don’t really believe it will close. You know, they’ve done four transplants, and all four have failed, so um …. But we’ve done everything, we’ve done the Pico, we’ve done the vacuum, we’ve done … pffff … I think I’ve tried everything at the hospital. (1109)

Fearing recurrences is related to the challenging experience of having a VLU and understanding the associated chronic conditions. This fear could be viewed as a motivator to engage in behavior change. One individual explains:

The fear is there, but I try to … it’s not … it’s not really a worry anymore. It’s a fear that I’m trying to … transform, and to take something positive from it, to use it as motivation to make sure it doesn’t happen again …. (3042)

Experiencing pain serves as a significant mediator of self-management strategies. Pain as an individual’s experience could impede the implementation of self-management strategies. One individual explained:

She’d given me … elastic bands to do exercises, to strengthen my calves, I continue to do my exercises at home, as best I can, given the pain I’m having because the second ulcer has spread to my ankle so … it’s not very pleasant for my calf. (1846)

However, for others, the experience of pain was the strongest motivator to change their behavior in order to avoid experiencing this symptom again: “I’ve decided to prevent ulcers from coming back after experiencing what it does to the patient; it hurts a lot, and I don’t want it to happen again” (3031).

These mediators of the process emerged during interviews and data analysis. However, as one participant argued: “We’re not robots yet, so we’re not all designed in the same way, and that’s where the problem lies when it comes to care techniques, because it is exactly that each human being needs to be analysed differently and individually” (1109), the existence of other mediators should be acknowledged.

### Living in a Conscientious Way

The conscientious integration of new strategies regarding VLU management in daily living entails adopting long-term changes and implementing new rituals adapted to one’s current daily life as an action to prevent recurrence or promote healing. This category encompasses the sub-category of having the responsibility and confidence in a better future. Experiences of change varied across individuals. Some individuals made minimal changes because their initial self-management strategies appeared effective: “So there hasn’t been much of a change, because until now we’ve realized that even in terms of nutrition, well, it was … it was good” (1809).

However, another individual changes his everyday practice when he goes to the food store. He further explains, “Now I’m much more careful, I look much more at the … the … the ingredients in the products I buy. I buy fewer industrial products too, and then … I prepare myself more …” (1807).

Integrating this new way of living improves regularity in self-management strategy and integrating them to the best they can in their daily life. Nevertheless, some participants highlight that they did not change following the intervention but that it opened their eyes in understanding why they should engage in self-management strategies. Understanding that VLUs result from a chronic condition meant that they should engage continuously. One individual explains, “As it’s a chronic disease that apparently can’t be cured, I’m going to have to keep going if I don’t want my condition to get any worse” (1807).

Having the responsibility refers to individuals’ accountability for their body. This included reflecting on their actions and their consequences with the aim to hold control: “And I have … hum … yes to remind myself that it’s myself who will play the most important role in the relapse, well … precisely in what I’m going to put in place” (3042).

This sub-category highlights the importance of shared responsibilities with healthcare providers and the need to be proactive in seeking early referral to a specialized center when experiencing a recurrence.

Confidence in a better future illustrates the need to envision a brighter future and the possibility of not experiencing VLUs too regularly. Individuals explain the significance of returning to the life they enjoyed before and utilizing the tools they have acquired to prevent recurrence. One individual expresses this as following: “So you see, I’ve had to make a lot of changes in … my little person to … precisely improve a healing … I hope uh … definitive of my ulcers” (1846).

### Theoretical Connections Between Categories

The overall category of being influenced by my own story significantly influences the whole decision-making process, either positively or negatively. Being personally informed supported the decision-making process and the adaptation of self-management strategies. This link was especially positive when educational sessions involve a meaningful relationship, relevant content, and appropriate modality. The conscientious decision-making process depends on various mediators which influence all positively or negatively the process and reflect decision making into unique circumstances. Progressively, the self-management changes were included into a conscientious way of living. However, some back–forward links are present as the decision to engage in self-adapted management strategies or living in a conscientious way needed individuals to reassess and realign their decisions and strategies according to the evolution of the mediators.

## Discussion

This co-constructed theory illuminates multiple realities of the complex process of decision making involved in engaging in self-management strategies following an interprofessional tailored nurse-led patient education program. To our knowledge, this CGT is the first theory explaining how individuals with VLUs experience an interprofessional tailored nurse-led education program and its repercussions regarding self-management. In doing so, the theory provides understanding of the existing gap regarding effectiveness of patient education programs for persons living with a VLU. While older systematic reviews did not describe the benefits of patient education for VLU wound healing or preventing recurrence (Shanley et al., 2020; Weller et al., 2016), the latest evidence suggests slight improvements in outcomes such as wound healing and wound size reduction, as well as other outcomes such as quality of life, pain, and functional ability (Bossert et al., 2023). According to our theory, this could be explained by the multiplicity of factors that either support or impede the implementation of self-management strategies and their possible adaptation by individuals in their daily routine. Qualitative findings suggest that various factors could either enable or hinder the use of compression therapy or exercises for individuals living with a VLU (Perry et al., 2023; Qiu et al., 2022). With the exception of unclear advice from healthcare providers, which was addressed by the tailored nurse-led intervention, most of their findings could be integrated into the mediating factors of our proposed theory. Nevertheless, considering the variability within the categories of our CGT, it seems important to acknowledge the decision-making process and understand why individuals either engage, adapt, or disengage in self-management strategies on an individual basis.

Pedagogical modalities and the content of interventions for individuals living with a VLU vary across existing evidence (Bobbink, Puglièse, et al., 2020). According to our CGT, findings suggests that using multiple pedagogical approaches and utilizing a multicomponent intervention in an individualized manner could benefit patients by enhancing their knowledge, skills, and self-management behaviors (Bolton Saghdaoui et al., 2023; Gomes et al., 2024). By its individually targeted approach, the intervention addresses the challenges posed by varying levels of health literacy, ensuring that each individual gained sufficient understanding of the disease to make conscientious decisions about whether to engage in self-management strategies. Offering a multicomponent intervention enables individuals to engage in adapted self-management strategies, providing them with the opportunity to make decisions about behavior changes that align with their everyday lives.

The findings from our study indicate that the efficacy of such an intervention cannot be evaluated exclusively based on wound size reduction, wound healing, or recurrences, given the unique aspects of patient education in individuals living with a VLU and in the challenges associated with fostering healing and prevention. In fact, due to the presence of associated diseases (Gethin et al., 2021) in this population, it is likely that components of the intervention, such as improving dietary habits or incorporating exercises, have greater overall benefits, as demonstrated in other populations (Kariuki et al., 2024; Kelly et al., 2014; Marquez et al., 2020). Van Hecke et al.(2013) highlight in a qualitative study involving 15 participants living with a VLU that knowledge deficits regarding etiology and treatment of VLUs were present in this population. Similar to Perry et al. (2023), these elements were not systematically observed in our results, as the level of knowledge varied greatly among participants in relation to their previous experiences with VLUs. Therefore, knowledge deficit could not be understood as a singular factor contributing to recurrence and underpin the importance of conducting individualized assessments to meet specific needs. Other research (Dhar et al., 2024; Van Hecke et al., 2011), in alignment with the proposed theory, supports the notion that individuals are more likely to consciously adhere to advice after receiving comprehensive information from experts, underscoring the significance of the relationship between individuals and healthcare providers (Rosenburg et al., 2023). Philips et al. (2018) discovered similarly robust findings regarding individual experiences of living with VLUs through a qualitative systematic review. Our CGT enhances this understanding by elucidating the connection between previous experiences and engagement in self-management. This connection is demonstrated by the flexibility inherent in each category, allowing for its uniqueness when interpreting individuals’ experiences through this theory.

Improving self-management through patient education for individuals living with a chronic disease has been a significant topic of discussion among healthcare providers for decades (WHO, 1998) leading to the development of various frameworks within or outside the nursing discipline. Bandura’s (1977, 1997) self-efficacy theory, renowned in behavioral science, refers to individuals’ confidence in their ability to organize and execute actions to achieve their goals under influence of sources of self-efficacy. Among these, “performance accomplishment” and “physiological state” align with the categories of our CGT, influenced by individuals’ own story and the mediator of attentiveness to the evolution of our CGT. Both highlight the positive impact of achievement in promoting engagement of VLU self-management. Bandura (1977, 1997) described “verbal persuasion” as a main influencer which was evidenced through the categories of “Being personally informed” or “Being supported by healthcare systems,” illustrating the importance of positive feedback or being encouraged from the nurse during different meetings.

For many years, models have promoted the benefits of self-care, which have sometimes influenced nurse-led patient education for individuals with VLUs (Herber et al., 2008). Additionally, our model emphasizes the importance of open communication between healthcare providers and individuals living with a VLU. Our model incorporates aspects of King’s (1981) conceptual model, highlighting mechanisms of goal setting and supporting as Klein et al. (2021) the significance of a meaningful relationship between healthcare providers and patients living with a VLU within a specific context. Therefore, and in alignment with Fawcett (2020), we critique the use of terms such as adherence, compliance, or concordance in this specific population.

Our proposed theory aligns well with the recent guidance provided by the WHO (2023) on patient education. By emphasizing the significance of multiple pedagogical interventions, the utilization of supporting materials, and effective communication to promote self-management, our proposed theory underscores the complexity of decision making and the promotion of good communication. Therefore, our model suggests acknowledging the conscientious process of decision making and focusing TPE as, in alignment with the Swiss Federal Office of Public Health (2022), an act to promote self-management strategies.

### Strengths, Limitations, and Quality Criteria in CGT

Grounded theories can offer valuable guidance for clinical practice (Nathaniel & Andrews, 2007). Therefore, this theory provides rich information to guide practice, offering healthcare providers a unique opportunity to understand processes underlying learning and implementation of self-management strategies in this specific population and context. Additionally, this theory will inform future research aiming at improving clinical outcomes for individuals living with a VLU. A strength of this study is the rigorous yet flexible approach used to explore the complex experiences of VLU individuals following tailored interprofessional nurse-led patient education and generating the theory grounded in the data. Nevertheless, this study posed some limitations. First, all the individuals included agreed to be involved in a clinical trial focusing on nurse-led patient education resulting in a selection bias. Second, the process of theoretical sampling did not allow us to include participants from the control group without impeding internal validity of the RCT, even though these individuals could provide meaningful insights of a “naïve” perspective on modalities and content of TPE suited for self-management of VLUs.

Pursuing quality in CGT is central (Charmaz & Thornberg, 2021) to enhance its use in practice. Therefore, different strategies were employed. Credibility was achieved through a rigorous methodology, discussion about methodological challenges in CGTM, and the inclusion of a diverse array of participants resulting in variety and richness of their storytelling. Verbatim quotes provide access to participants’ meanings and are presented throughout the manuscript. Resonance was attained through the coding process, discussion with participants and experts, and comparisons with evidence. This contextualized theory provides originality by offering new insights into an underexplored phenomenon, thereby providing understanding of a specific healthcare challenge. By facilitating further development of interventions to improve outcomes for individuals living with a VLU, this CGT will meet the criteria of usefulness.

## Conclusions

This study aimed to develop a theory to explain VLU individuals’ experiences of following a nurse-led patient education program regarding self-management. The theory “Conscientiously Engaging in Self-Management” was co-constructed between those living with VLUs and the research team. Future development of a patient education program should reflect the use of this theory to support patients facing negative mediators focusing on goal setting prior to adherence/compliance/concordance to therapy. More research is needed to improve persons’ involvement in self-management strategies and find robust outcomes to promote contextually appropriate evidence-based practice.

## Supplemental Material

Supplemental Material - Making Conscientious Decisions: Engaging in Venous Leg Ulcer Self-Management Following Nurse-Led Patient Education Supplemental Material for Making Conscientious Decisions: Engaging in Venous Leg Ulcer Self-Management Following Nurse-Led Patient Education by Paul Bobbink, Géraldine Gschwind, Philip Larkin, and Sebastian Probst in Qualitative Health Research
