# Supplementary material for: Making Conscientious Decisions: Engaging in Venous Leg Ulcer Self-Management Following Nurse-Led Patient Education
Source: Qual Health Res. 2024 Oct 28;35(8):901–15. doi: 10.1177/10497323241285692 (PMC12117134; doi:10.1177/10497323241285692)
Supplement: Supplemental Material - Making Conscientious Decisions: Engaging in Venous Leg Ulcer Self-Management Following Nurse-Led Patient Education [file sj-pdf-1-qhr-10.1177_10497323241285692.pdf]

Interview ID	Original verbatim in French	Translated English verbatim
1710	*Comme je dis toujours : on doit tous mourir un jour hein !*	*As I always say, we all have to die one day!*
2030	*Original interview in English*	*I reached a certain age, things happen and you just have to accept it, there is nothing else you can do.*
1807	*J'ai été toxicomane avant et puis j'ai mis des années à….arrêter de consommer de l'héroïne, donc ….euh….j'ai réussi à arrêter le jour ….où…je me suis dit, ben voilà maintenant c'est ou dans deux mois je suis « froid » ou je fais quelque chose. Avant je n'en voyais pas l'utilité non plus, et puis personne aurait pu me faire arrêter… Avant les gens auraient pu me dire n'importe quoi je n'aurais pas arrêté quoi .*	*I used to be a drug addict and it took me years… to stop using heroin, so ....hum....I managed to stop on the day ....when...I said to myself, well now it's either in two months I'm "cold" or I'm doing something. Before I didn't see the point either, and then no one could made me stop... Before people could have told me anything I wouldn't have stopped anything.*
3012	*Pour moi un ulcère, c'est interne, c'est dans l'estomac.*	*For me an ulcer is internal, it's in the stomach.*
2030	*Original interview in English*	*The thing is, first of all, what I have, and what I am going through, I didn't know that this exists…[…] So I had a shock to know I found myself in such a situation where I know nothing about.*
3017	*Parce qu'avec avec le temps je ne veux pas dire que je suis expert mais je me rends compte de deux ou trois trucs.*	*Because with time, I don't want to say that I'm an expert, but I realise two or three things.*
1070	*Il aura fallu que j'aie ce genre de …de…de rencontre pour avoir des réponses à des questions vraiment très simples en fait ; comme : est-ce que c'est utile de marcher ? Ou est-ce que c'est*	*I had to have this kind of ...of…of meeting to get answers to some really simple questions, like: is it useful to walk? Or is it bad for the wound?*

	mauvais pour la plaie ?	
1809	*Mais en plus quand quelqu'un connaît [les ulcères] et qu'on peut parler ben ça soulage quand même.*	*But what's more, when someone knows about [ulcers] and you can talk with, well that's a relief.*
3017	*Bon après maintenant on a réussi à s'apprivoiser et maintenant on s'adore.*	*Well, now we have managed to tame each other and now we adore each other.*
3017	*Je pense qu'avec elle ça va bien parce que ça va dans les deux sens donc elle me propose des trucs et moi je je enfin la communication va dans les deux sens donc elle me dit des trucs je les intègre et je lui dit euh euh enfin je refuse pas de faire des choses, mais disons je lui je lui apporte aussi un feedback sur ce que je vois.*	*I think that things are going well with her because it's a two-way process, so she suggests things to me and I, I, well, communication is going in a two-way direction, so she tells me things, I take them on board and I tell her hum, hum, well, I don't refuse to do things, but let's say I give her feedback on what I see.*
3028	*Elle m'a expliqué le problème qui se trouve chez moi, avec des petits mots que je peux comprendre. Parce que pour moi, tous ces termes c'était pour moi, au début, je trouvais difficile, je ne comprenais pas ….le message qu'ils voulaient me transmettre. Mais après j'ai bien compris, elle m'a expliqué avec …Mais vraiment avec des dessins.*	*She explained to me the problem I was having, in little words I could understand. Because for me, all these terms were, at first, difficult, I didn't understand .... the message they wanted to convey. But then I understood, and she explained it to me with...but really with drawings.*
3012	*C'est une bible dans …dans son contexte.*	*It's a bible in … in its context.*
1070	*Sans vouloir me désolidariser de la pertinence de cette brochure qu'en fait elle s'adresse surtout à des gens beaucoup plus âgés que moi…et puis…et*	*Without wishing to dissociate myself from the relevance of this brochure, in fact it is mainly aimed at people much older than me... and then... and then that's it, I just had a look.*

	puis voilà, moi j'ai juste jeté un œil.	
3031	*Ça ce sont les trois éléments qui m'ont véritablement impressionné dans cette brochure, c'est cette approche systémique complète et extrêmement pratique d'ailleurs, qu'on peut mettre en œuvre après quelques explications ….qui m'ont été données justement par [infirmière d'étude] et son équipe.*	*These are the three elements that really impressed me in this brochure, this complete and extremely practical systemic approach, which can be implemented after a few explanations .... which were given to me by the [study nurse name] and the team.*
3042	*Ben pour moi, déjà, j'ai appris aussi des choses, euh….pour moi personnellement ce qui est important, c'est maintenant de…..euh…..appliquer ce que je savais déjà et ce que j'ai appris lors de cette étude euh…en terme de prévention pour pas qu'il y ait à nouveau une rechute, enfin que je refasse à nouveau une plaie.*	*Well, for me, I've already learnt a few things, um.... for me personally, what's important now is to.....hum.....apply what I already knew and what I learnt during this study um...in terms of prevention so that there isn't another relapse, well that I don't have another wound.*
3012	*Ben si vous me dites qu'il faut les mettre tous les jours, ben on les mettra tous les jours, si vous me dites qu'il faut les mettre un jour sur deux, on les mettra un jour sur deux. Chu bête et discipliné.*	*If you tell me you have to wear them every day, well I will wear them every day. If you tell me you have to wear them every two days, we'll I will wear them every two days. I am stupid and disciplined.*
3017	*Bon avec moi il faut planter la graine et après un moment elle pousse.*	*Well, with me you have to plant the seed and after a while it grows.*
1809	*On n'a pas le choix, la meilleure chose c'est de faire ce qu'on doit faire au moment où il faut le faire, mais de temps en temps se permettre quand*	*I don't have a choice, the best thing is to do what you have to do when you have to do it, but from time to time allow yourself a few liberties.*

	même quelques libertés.	
1070	*J'ai fait déjà des efforts sur d'autres plans, j'ai de la peine à fournir tous les efforts en même temps, voilà c'est aussi ça le problème.*	*I've already made efforts in other areas, but I'm finding it hard to make all these efforts at the same time, and that's part of the problem.*
1807	*Bon il y a aussi les bas de contention, après je sais que je devrais logiquement porter des bas de contention euh… à vie, ça j'ai beaucoup de peine à accepter aussi…. Et puis …normalement je devrais mettre- j'ai la plaie à la jambe droite, et puis j'ai eu aussi il y a plusieurs années euh… mais qui s'est refermée toute seule à la jambe gauche et puis je devrais mettre un bas aussi à la jambe gauche, mais ça j'ai….ça…euh…. j'ai beaucoup de peine aussi à le faire.*	*Well there are also the compression stockings, after all I know that logically I should be wearing them for life, which I also found very hard to accept.... And then ... normally I'd have to wear - I've got a wound on my right leg, and then several years ago I also had a wound on my left leg, which closed up by itself, and then I'd have to wear a stocking on my left leg as well, but that's something I find very hard to accept too.*
1710	*Disons….parce qu'après, disons ….j'ai…le problème c'était que j'avais ….j'avais une attelle que j'avais eu presque un mois voyez ?! Donc je ne pouvais plus bouger autant qu'avant et puis euh…donc les exercices, donc je les faisais disons avec l'attelle, du côté gauche….je faisais droite, gauche, euh…en avant en arrière, et puis …bon je pouvais pas forcer comme …comme j'aurais voulu,*	*Let's say ....I...the problem was that I had a ....I had a splint that I'd had for almost a month, you see!? So I couldn't move as much as I used to and then um...so the exercises, so I did them let's say with the splint, on the left side....I did right, left, um...forwards and backwards, and then ...well I couldn't force it like ...like I would have liked…*
1070	*Et manger très sainement c'est une activité à part entière hein…si vous me permettez.*	*And eating very healthy is an activity in its own right… if you don't mind me saying so.*
3012	*Si j'aurais deux fois plus de temps, là on va*	*If I had twice as much time, we'll I would chop the vegetables, cook them,*

	commencer à dire ben on va couper les légumes, les cuire, machin …et ci et ça. Mais là franchement, euh…non on n'a pas trop le temps…	*and so on and so forth. But honestly, hum... no, we don't have much time....*
3042	*Mais en tout cas ça m'a apporté le fait de me re-réveiller un peu à ce niveau-là, que cela vaut vraiment la peine de faire attention et….et c'est important même si c'est pas visible tout de suite ou….ça a son importance dans la ….pour la peau quoi.*	*But in any case, it's given me a wake-up call in that respect, that it's really worth paying attention and....and that's important even if it's not immediately apparent... but it has its importance in the….for the skin.*
1807	*Ben au boulot par exemple, dans les…automates à boisson, on a que des limonades…on a que des limonades avec 10 milligrammes de sucre par décilitre, donc il n'y a rien d'autre.*	*Well at work, for example, in the drink machines, you only get lemonade...you only get lemonade with 10 milligrams of sugar per deciliter... so there is nothing else.*
1109	*Ça il faut garder son indépendance, et il faut …il faut pas non plus se laisser aller à rester avachi toute la journée dans son fauteuil toute la journée.*	*You have to keep your independence, and you must.. you mustn't let yourself slump in your armchair all day either.*
1809	*Malheureusement certains exercices je ne peux pas faire comme tout le monde parce que j'ai eu deux accidents sur 2018…donc en plus je n'ai plus de tendon dans les bras, donc ça veut dire que je suis quand même handicapée pour pas mal de choses au niveau des bras et qui ne me permet pas de tirer l'élastique ou de faire ça, mais par contre tout ce que je peux faire au niveau des chevilles, des*	*Unfortunately, I can't do some exercises like everyone else because I've had two accidents...so on top of that I no longer have any tendon in my arms, so that means I'm still handicapped for quite a few things in my arms and that doesn't allow me to pull a rubber band or do that, but on the other hand everything I can do with my ankles, knees, feet and all that, I showed her [the nurse], she told me that I proceed correctly.*

	genoux, les pieds tout ça, je lui ai montré [l'infirmière], elle m'a dit que c'était juste.	
1809	*Ce n'est pas comme si j'avais la possibilité de vivre sans mettre de de bas de contention pour la simple et bonne raison que ce n'est pas vivable dans ces conditions, parce que je pense qu'elle vous a dit que j'avais le syndrome de Leiden c'est pour ça non.*	*It's not as if I could live without wearing compression stockings, for the simple reason that it's not bearable. Because I think she [the study nurse] told you I have Leiden syndrome that's why no.*
2050	*Je vois déjà par rapport à si on peut manger un peu de tout c'est déjà la cicatrisation se passe beaucoup mieux se fait beaucoup mieux.*	*I can already see that if you can eat a bit of everything, the healing process improve, going much more smoothly.*
1109	*Mais moi je n'y crois pas trop. Vous savez ils ont fait quatre greffes, et les quatre ont loupé, donc euh….Mais on a tout mis, on a mis le pico, on a mis le vacuum, on a mis…pffff…Je crois que j'ai tout essayé à l'hôpital*	*But I don't really believe it will close. You know, they've done four transplants, and all four have failed, so um.... But we've done everything, we've done the Pico, we've done the vacuum, we've done...pfffff...I think I've tried everything at the hospital*
3042	*La peur est là quoi, mais j'essaie de la….c'est pas …….c'est….c'est plus vraiment une angoisse comme ça, c'est une peur que j'essaie de …transformer, et d'en prendre du positif justement, de prendre comme motivation pour faire en sorte que cela n'arrive plus.*	*The fear is there, but I try to...it's not … it's not really a worry anymore. It's a fear that I'm trying to ... transform, and to take something positive from it, to use it as motivation to make sure it doesn't happen again.*
1846	*Elle m'avait donné des…élastiques pour faire des exercices, pour renforcer les mollets que je continue de faire à la maison, le mieux que je peux d'après les douleurs que j'ai parce que le deuxième*	*She'd given me...elastic bands to do exercises, to strengthen my calves, I continue to do my exercises at home, as best I can, given the pain I'm having because the second ulcer has spread to my ankle so... it's not very pleasant for my calf.*

	ulcère s'est placé sur la cheville, donc… ce n'est pas très agréable pour mon mollet.	
3031	*Je suis décidé à prévenir le retour des ulcères après avoir expérimenté ce que cela fait au patient ; ça fait très mal, et puis je n'ai pas l'envie du tout que ça recommence.*	*I've decided to prevent ulcers from coming back after experiencing what it does to the patient; it hurts a lot, and I don't want it to happen again.*
1109	*On n'est pas encore des robots, donc on n'est pas tous conçu pareil, donc c'est là où le bas blesse un peu dans la technique des soins, c'est justement que chaque être humain doit être analysé différemment et individuellement…*	*We're not robots yet, so we're not all designed in the same way, and that's where the problem lies when it comes to care techniques, because it is exactly that each human being needs to be analysed differently and individually...*
1809	*Alors, il n'y a pas eu grand-chose comme changement, parce que jusqu'à présent on s'est rendu compte que même au niveau nutrition, ben c'était le…c'était bon.*	*So there hasn't been much of a change, because until now we've realized that even in terms of nutrition, well, it was...it was good.*
1807	*Maintenant je fais beaucoup plus attention, je regarde beaucoup plus les …la…les ingrédients qu'il y a dans les produits que j'achète, j'achète moins de produits euh….fabriqués industriels aussi, et puis…je me prépare plus….*	*Now I'm much more careful, I look much more at the ...the...the ingredients in the products I buy. I buy fewer industrial products too, and then...I prepare myself more....*
1807	*Comme apparemment c'est une maladie chronique qu'on n'arrive pas à guérir, donc je serai bien obligé de continuer si je ne veux pas que…ma condition empire quoi.*	*As it's a chronic disease that apparently can't be cured, I'm going to have to keep going if I don't want my condition to get any worse.*

3042	*Puis je peux ….euh….oui de me rappeler que c'est moi-même qui va jouer le rôle le plus important pour la rechute, enfin ….dans justement ce que je vais mettre en place.*	*And I have… hum … yes to remind myself that it's myself who will play the most important role in the relapse, well .... precisely in what I'm going to put in place.*
1846	*Alors vous voyez, j'ai dû faire plein de changements dans dans…ma petite personne pour…justement améliorer une guérison…j'espère euh….définitive de mes ulcères.*	*So you see, I've had to make a lot of changes in...my little person to...precisely improve a healing...I hope uh....definitive of my ulcers.*
